# Musical hallucinations and their relation with epilepsy

**DOI:** 10.1007/s00415-019-09289-x

**Published:** 2019-04-10

**Authors:** J. A. F. Coebergh, R. F. Lauw, I. E. C. Sommer, J. D. Blom

**Affiliations:** 1 Department of Neurology, Haga Hospital, The Hague, The Netherlands; 2 Department of Neurology, Ashford and St. Peter’s Hospital, Chertsey, UK; 3 Department of Neurology, St. George’s Hospital NHS Foundation Trust, Tooting, London, England UK; 4 Parnassia Psychiatric Institute, The Hague, The Netherlands; 5 Department of Psychiatry, University of Groningen, Groningen, The Netherlands; 6 Faculty of Social and Behavioural Sciences, Leiden University, Leiden, The Netherlands

**Keywords:** Antiepileptic, Auditory hallucination, EEG, MEG, Musical hallucinosis, Pharmacotherapy

## Abstract

Musical hallucinations are poorly understood phenomena. Their relation with epilepsy was first described over a century ago, but never systematically explored. We, therefore, reviewed the literature, and assessed all descriptions of musical hallucinations attributed to epileptic activity. Our search yielded 191 articles, which together describe 983 unique patients, with 24 detailed descriptions of musical hallucinations related to epilepsy. We also describe six of our own patients. Based on the phenomenological descriptions and neurophysiological data, we distinguish four subgroups of epilepsy-related musical hallucination, comprising auras/ictal, inter-ictal and post-ictal phenomena, and phenomena related to brain stimulation. The case descriptions suggest that musical hallucinations in epilepsy can be conceptualised as lying on a continuum with other auditory hallucinations, including verbal auditory hallucinations, and—notably—tinnitus. To account for the underlying mechanism we propose a Bayesian model involving top-down and bottom-up prediction errors within the auditory network that incorporates findings from EEG and MEG studies. An analysis of phenomenological characteristics, pharmacological triggers, and treatment effects suggests wider ramifications for understanding musical hallucinations. We, therefore, conclude that musical hallucinations in epilepsy open a window to understanding these phenomena in a variety of conditions.

## Introduction

Between musical hallucinations and epilepsy a connection exists that was already suspected when neurology was still struggling to comprehend either phenomenon. In 1883, Ormerod wrote an article in Brain, entitled *On epilepsy, in its relation to ear-disease*, in which he mused that, ‘it needs no very extended observation to show that these two phenomena frequently co-exist’. In this pioneering paper, he described a 61-year-old woman with longstanding deafness and epilepsy. The deafness progressed with increasing tinnitus, and gradually the woman also began to hear words, names, street sounds, and songs. Ormerod quoted Schwartze, who had said that, ‘subjective aural sensations, which are caused by demonstrable affections of the ear, may in predisposed persons (...) become the direct cause of aural hallucinations’ [1]. Well over a century later, the relation between epilepsy and musical hallucinations still puzzles us. Musical hallucinations are auditory hallucinations characterised by songs, tunes, melodies, harmonics, rhythms, and/or timbres [2]. They are different from earworms or ‘tunes in the head’, in the sense that they are perceptual in nature, whereas earworms are not. First described by Baillarger in 1844, musical hallucinations have traditionally been considered rare [3]. However, several studies in designated populations suggest that they are probably merely underreported. In a large sample (*n* = 1007) of patients attending an audiology clinic, for example, auditory hallucinations were common in the last four weeks (*n* = 114), with musical hallucinations (*n* = 52) increasing in frequency with increasing hearing loss, and correlating with tinnitus and female sex [4]. Musical hallucinations are commonly divided into two groups. When they occur in the absence of any associated pathological abnormalities—with the exception of hearing impairment (hypoacusis)—they are called idiopathic musical hallucinations. When they occur in the presence of associated pathological abnormalities, they are called symptomatic musical hallucinations [5]. The most relevant etiological factors for musical hallucinations are brain injuries, epilepsy, psychiatric disorders, and the use of illicit substances or medicines [6]. Recent insights into the neurophysiology of musical hallucinations, as well as the application of Bayesian prediction models, provide a window on these complex phenomena, and allow us to catch a glimpse of their pathophysiology [7, 8].

### Demarcating the area

Epilepsy and musical hallucinations can be related in several ways, with the latter presenting as an aura/ictal phenomenon, or a post- or interictal phenomenon. Moreover, both conditions can be experienced as a side effect of the same medication or share some other common etiology (e.g., a structural lesion) and/or co-morbidity (e.g., psychiatric disease). Importantly, the co-occurrence of epilepsy and musical hallucinations should be differentiated from musical reflex epilepsy (where music in the external world triggers a seizure) and cases where an actual relation between the two phenomena is uncertain. In a series of 666 cases with temporal-lobe epilepsy, for example, in which 16% of the patients experienced auditory hallucinations, the great majority of the hallucinations were unformed (e.g., banging, ticking) and there was no documentation of musical hallucinations [9]. In other studies, complex auditory hallucinations characterised by well-formed and—occasionally quite specific—auditory symptoms, such as music and well-defined human voices, were reported by 21 out of 53 patients with idiopathic partial epilepsy [10], whereas, in another study, nine out of 352 patients with generalised epilepsy experienced auditory verbal hallucinations [11]. Sometimes musical hallucinations have been ascribed to epilepsy when there were EEG abnormalities (not necessarily correlating with the clinical phenomenon) and/or a response to antiepileptics (i.e., pharmacological dissection); which is both questionable evidence of a relation. In another study, three out of 16 individuals with musical hallucinations (17.6%) had changes in the EEG (two with epileptiform activity in the left temporal region or in the midline/bilateral parasagittal region, and one with slow waves at the posterior temporal/left occipital regions), even though none of them exhibited symptoms of epileptic seizures [12]. A final example stems from Wengel et al. [13] who noted that three out of their five patients with musical hallucinations showed temporal-lobe abnormalities on the EEG (one slow, one sharp-wave, and one alpha and theta transients).

### Aim of the present review

As will be clear from these examples, and from the fact that the interobserver variability in EEG reporting and the lack of blinding are difficult to overcome, the purported relationship between EEG abnormalities and musical hallucinations is often difficult to establish with certainty. However, there are also cases where there is a clear and unambiguous relationship. In the present review, we critically appraise this type of literature on epilepsy and musical hallucinations, and place our findings in the context of models of magnetoencephalography (MEG) changes of (de-)synchronisation [7, 8, 14]. In addition, we borrow from the field of network science in relation to tinnitus to generate further hypotheses about the pathophysiology of epilepsy-induced musical hallucinations, and investigate whether these mechanisms can be generalised to other types of musical hallucination.

## Materials and methods

For the purpose of this review, we searched the databases of MEDLINE, PsycINFO, and Embase Psychiatry for articles matching the terms music* hallucin*, and musical hallucination, and added relevant papers from our personal collection with titles such as ‘Musical ear syndrome’, ‘Auditory Charles Bonnet syndrome’, and ‘The sound of silence’. We examined all articles published between 1890 and August 2017 that were written in English, Dutch, French or German. We excluded all reports of musical illusions, palinacusis, hypnagogic or hypnopompic hallucinations, and obsessions (i.e., ‘earworms’). Incidentally, our interpretation of obsession versus hallucination (i.e., ‘earworm’ versus musical hallucination) rarely differed from that of the original authors (e.g., Islam et al. [15]). Epilepsy was deemed the main cause when the onset of musical hallucinations was temporally linked to a seizure, e.g., an aura/ictal episode or post- or interictal episode, when the hallucinations improved with surgical treatment for likely epileptic foci, and/or when epileptiform EEG abnormalities disappeared which had been present prior to treatment (in the appropriate clinical context). Musical hallucinations that occurred post-ECT or with other types of brain stimulation were also included since their pathophysiology appears to be quite similar. In our review, we included patients we have seen as part of our ongoing natural-history study. They gave informed consent to be included in the study. The study was approved by the Local Research Ethics Committee, and performed in accordance with the ethical standards laid down in the 1964 Declaration of Helsinki and its later amendments.

## Results

**Fig. 1:**
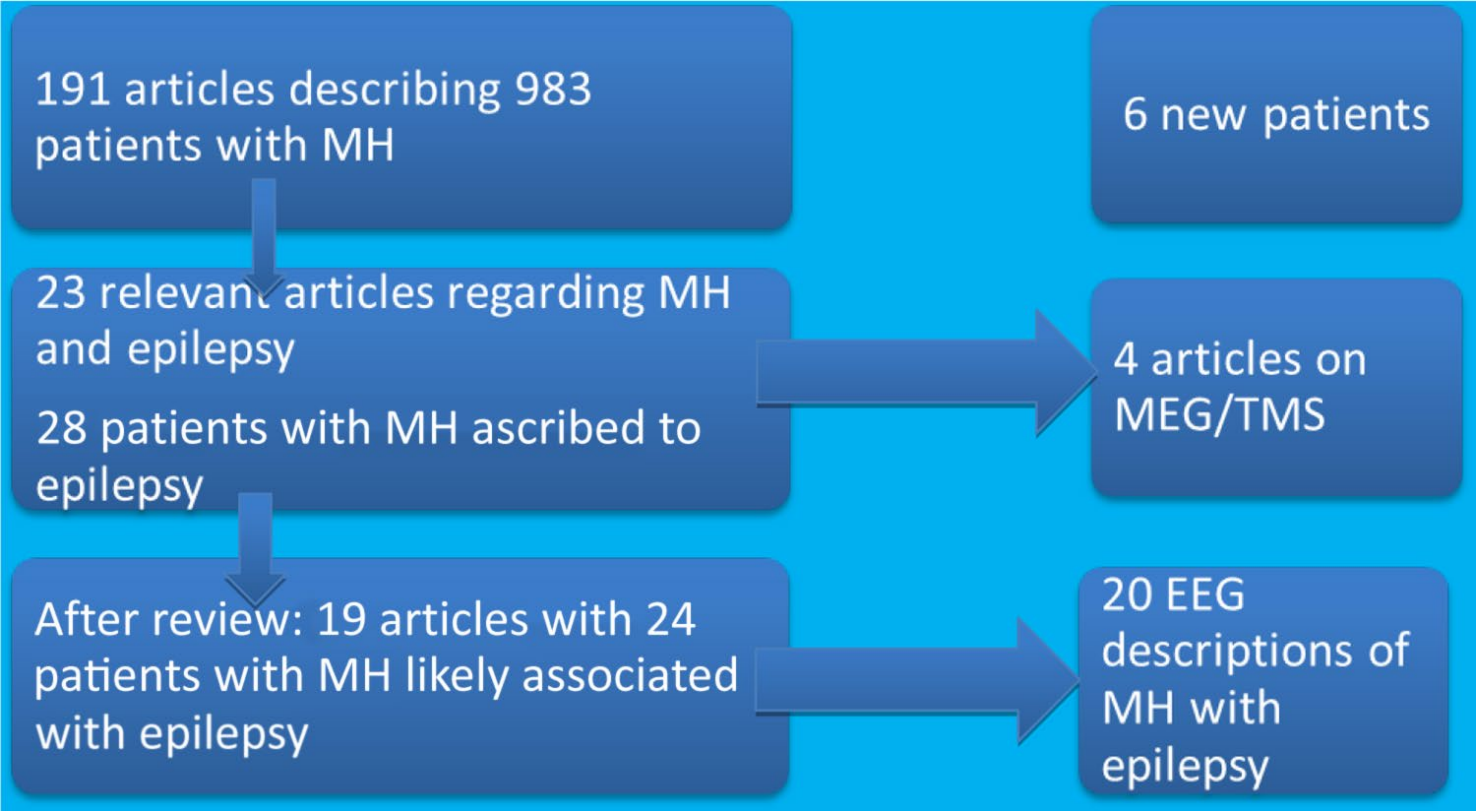
Flowchart for article and patient selection

Our literature search yielded a total number of 214 articles, of which 191 were considered eligible for inclusion. Together, they discussed 983 unique patients (see flow chart, Fig. 1). Among this collection were various modest case series, including one which described 44 patients with musical hallucinations in the context of alcohol hallucinosis, one which described the lifetime presence of musical hallucinations in 55 patients diagnosed with obsessive–compulsive disorder (OCD) and/or schizophrenia spectrum disorder [16, 17], and one very large case series of 393 on musical hallucinations and brain disease [18]. In separate case studies, 24 patients with epilepsy as a possible cause were described with some detail (Table 1). However, it was not always possible to obtain full descriptions from the original papers. For example, Golden and Josephs alluded to 16 cases of musical hallucination related to epilepsy, of which they described only one in detail [18]. Most of the cases described in the literature were considered ictal or post-ictal in nature, and either resolved spontaneously, or tended to respond well to antiepileptics or surgery for the underlying cause [19–21].

**Table 1:** Summary of clinical findings

Article	Age/sex	Clinical diagnosis	Clinical presentation	EEG findings	Treatment
*Aura and ictal*					
Wilson [24]			Aura		
Rennie [19]	M	Left temporal glioma	Aura	Left temporal spikes	Surgery and AED
Ozsarac et al. [25]	50M	Left temporal AVM	Music and other auditory hallucinations, aura	NA	No treatment
Golden and Josephs [18]	33F	Right superior temporal astrocytoma	Focal epilepsy involving musical aura	NA	NA
Coebergh et al. (present paper)	19F	MRI Splenium abnormality after childhood meningitis	Aura once	Photosensitivity and interictal multifocal general spike/wave on EEG	AED: better
	39M	Left MCA stroke 7 years previously	Aura	Fast beta and theta intermixed centrally	AED: better
Penfield and Erickson [26]	38F	Left temporal gliosis	Ictal	NA	
Penfield and Perot [27]	16F	No lesion seen during surgery	Ictal		
Mulder and Daly [33]	40M	Left temporal lobe tumour	Music “like angel voices” during seizure	NA	NA
Schiffter and Straschill [35]	35F	Old right temporal-parietal bleed and epilepsy	Post-surgery 10 days reducing frequency and complexity	Left temporal rhythmical alpha and spike/sharp waves with musical hallucinations only	Resolved
Wieser [21]	22F	Asymmetric atrophy right Sylvian fissure	Ictal	Right temporal gyrus of Herschl discharge (intracranial)	Surgery
Sacks [31]	88F	Acute stroke right temporal lobe	Ictal	Spike/sharp wave bitemporal during musical hallucinations	AED (+/- time)
	80F	Hearing loss	Ictal	Sharp/spike-like waves during musical hallucinations	AED: full response
	M	Glioma	Ictal	‘Frankly convulsive’	No treatment
Fénelon et al. [30]	58F	Left parietal metastasis	Ictal	Abnormal, no paroxysms	Surgery
Isaacson et al. [36]	60M	Cholesteatoma right side; intracranial normal	Ictal/post-ictal	Right temporal 90 s 10-11 Hz sharp rhythmic activity - focal slowing - return to normal	AED (and time/ stopping ciprofloxacin)
Roberts et al. [20]	61F	Aneurysm pressing on right uncus; hearing normal	Ictal?	Rhythmic spikes locally on corticography	Surgery
Gentile et al. [28]	45F	MRI normal; PTA normal	Ictal; specific song by a popular Italian artist 1-3/30 with and without abdominal pain	Continuous spikes/sharp-wave complexes left mid-temporal region, spreading to anterior temporal, parietal, and frontal	Carbamazepine
de Maeseneire et al. [32]	64F	Left insular-temporal tumour	Attacks of known music; likely ictal	PLED during musical hallucinations left frontotemporal	AED: resolved
Martinez-Perez et al. [34]	45M	Left temporal-parietal glioma; right hearing loss	Ictal	Left frontal/temporal spikes and sharp waves	AED: resolved
Coebergh et al. (present paper)	34M	Left temporal glioma	Ictal; likely seconds	NA	Resolved after surgery
*Post-ictal/* *Interictal*					
Nielsen and Jacobs [75]	39M	Asymmetric plantar response	Post-ictal (many attacks)	‘Petit mal epilepsy’	
Hécaen and Ropert [38]	48F	Old left temporal traumatic brain injury and hearing loss	Post-ictal (once)	Slow left temporal (unknown date after musical hallucinations)	
Rennie [19]	M	Cysticercosis and hearing loss	Post-ictal six days	Normal (later)	NA
Donnet and Régis [29]	81F	MRI: atrophy and white-matter change; bilateral hearing loss; depression	Inter-ictal/post-ictal two weeks constant	Left temporal sharp and desynchronisation during musical hallucinations; normal at other time	AED: resolved
Couper [37]	78F	Old right occipital stroke and hearing loss	After generalised tonic/clonic seizure continuous musical hallucinations for 34 days	Sharp waves left posterior temporal (previously normal)	Change of AED
Borelli et al. [39]	62F	Right hippocampal-temporal sclerosis; hearing normal	Interictal persistent	Normal during musical hallucinations	Not bothersome
Coebergh et al. (present paper)	51F	Old right occipital/temporal ICH	Post-ictal two days	Day 4 right temporal-occipital delta burst	Resolved
	50M	Old right parietal stroke	3 years after onset focal seizures; interictal?	Right temporal epileptiform and sharp waves	Resolved
	70F	Epilepsy	Post-ictal 2-3 days	NA	Resolved

*NA* not available, *AED* anti-epileptic drugs, *PTA* pure tone audiometry

### Musical hallucinations as aura phenomena

Epileptic auras of a musical nature are rare. In a pathophysiological sense, auras are considered ictal processes. Nonetheless, since their phenomenology is different from classic ictal phenomena, and in the original papers they are often designated as ‘auras’, we decided to set them apart here for the sake of clarity. In total, we found four unique patients described in the literature. In two large—and rather old—case series, they were noted in 1881 by Gowers in two out of 1450 patients with epilepsy, and in 1933 by Lennox and Cobb in two out of 1359 patients [22, 23]. A lively impression of such an aura was rendered by Wilson [24], who quoted his patient as saying, ‘The aura took the form of a voice at a distance singing ‘Roses of Picardy’, distinct though faint; gradually coming nearer, the last notes seemed loudest of all; thereafter came a sense of terror, loss of speech and unconsciousness’. Rennie described a patient with a rather stereotyped epileptic aura, where the song Happy Talk from the film South Pacific would ‘come into his mind’ without warning [19]. After about seven bars, during which the music would become louder when he turned his head to the left (although he did not localise the music to either ear), his mind would seem to drift, and he would be unable to speak for a few seconds, after which he would be perfectly normal again. Rennie’s patient mentioned that sometimes he could abort the attack by bowing his head. Playing his own record of the sound track never caused or aborted an attack. At surgery a left temporal glioma was found, which was held responsible for the aura phenomena, including the hallucinated music. Ozsarac et al. [25] described a patient who for 10 years had had auras before most of his seizures, which would consist of vivid auditory hallucinations of conversations with friends, dialogues from movies, and songs of his favourite groups. The song by Pink Floyd, Another Brick in the Wall, Part 2, was a frequent auditory hallucination preceding an epileptic seizure. A large AVM occupying the left temporal lobe and part of the parietal lobe was held responsible for this. Golden and Josephs, in their large case series, mention a case of focal epilepsy involving musical aura secondary to a right superior temporal pilocytic astrocytoma [18].

We ourselves saw a 39-year-old man with repeated brief episodes of hearing music (he could not specify) before becoming unresponsive with hand rubbing for a minute, followed by brief disorientation, who had suffered a left MCA stroke 7 years before. His interictal EEG showed fast beta waves in fronto-central areas and theta waves intermixed centrally. We also saw a 19-year-old woman who several times in one evening heard music of the band Oasis, lasting for a few minutes each time, upon which she went downstairs to check where it came from, finding no source. Later that evening, she suffered a generalised seizure. The MRI head showed a stable splenial hyperintensity, probably due to childhood meningitis. Her interictal EEG showed multifocal epileptiform discharges.

### Ictal musical hallucinations

In the literature reviewed, ictal phenomena constituted most of the epilepsy-related musical hallucinations. From the wealth of case descriptions, 15 were sufficiently detailed. In what follows, we will render some of the most striking reports. One classic description stems from Penfield and Erickson, who treated a woman of 38 who 2 years after the removal of a meningioma arising from the left lesser wing of the sphenoid began to have attacks during which she heard voices coming from the right, and—occasionally—music or singing [26]. Even though she could move about and see, she was unable to hear anything during the attacks, except for the hallucinations. She was eventually cured by the removal of some scar tissue at the tip of the temporal lobe. In another paper, Penfield and Perot described a girl of 16 who had had attacks during which she would hear her mother sing Hushaby My Baby [27]. Meanwhile there was often a dream-like feeling of being in church or in a convent. When Brodmann areas 41–42 (posterior portion of the first temporal convolution in the auditory field) were stimulated, she said, ‘I hear music now, a funny little piece…’. Gentile et al. [28] described a 45-year-old woman with a 10-year history of abdominal pain attacks occurring one to three times per month, who in the preceding 5 years had experienced the melodies and lyrics of a song by a popular Italian artist. The song tended to start abruptly, and was generally heard immediately after the abdominal attacks, although occasionally in the absence of any pain. During one of those attacks, the EEG showed continuous spikes and sharp-wave complexes beginning in the left mid-temporal region, after which they spread out to anterior temporal, parietal, and frontal regions. After a 6-month period of treatment with carbamazepine (gradually titrated up to 800 mg/day) the abdominal pain attacks as well as the musical hallucinations disappeared. When after 1 year without attacks she reduced the use of carbamazepine to 400 mg/day, the episodes came back. Upon returning to 800 mg/day there was no further recurrence of abdominal pain or musical hallucinations over the subsequent year.

**Fig. 2:**
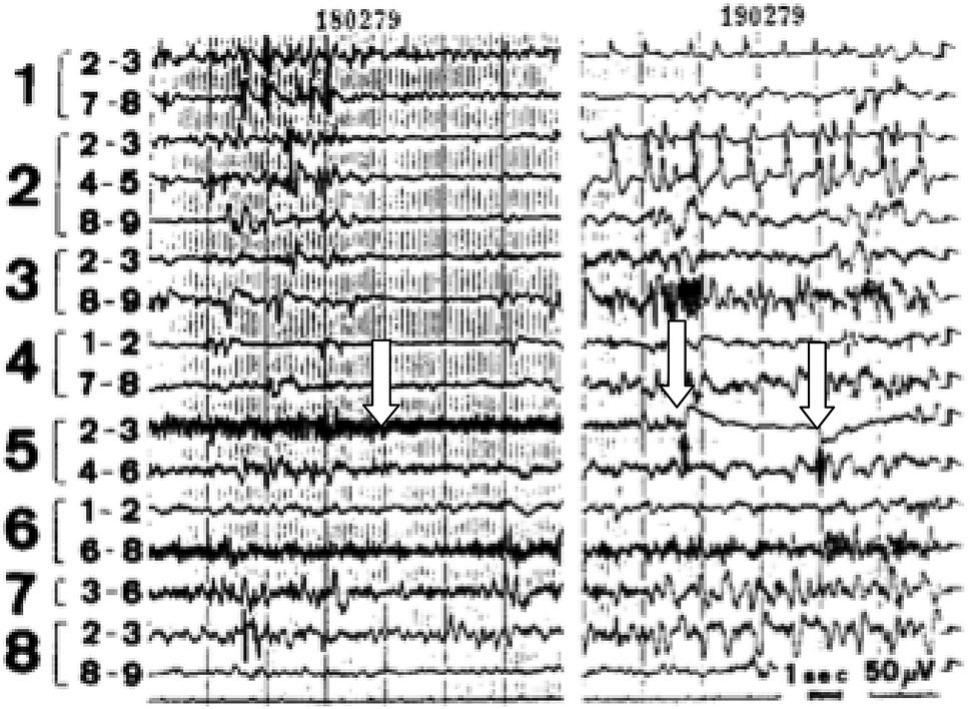
Intracranial EEG showing seizure patterns concomitant with musical hallucinations: the spontaneous discharges in the gyrus of Heschl could be controlled only by very elaborate auditory stimuli. Left: the beginning of a Portuguese song changes the frequency of the epileptic discharge (see arrow). Right: a sudden interruption of the song (between arrows) suppressed and modified the otherwise continuous discharge (Wieser [21])

**Fig. 3:**
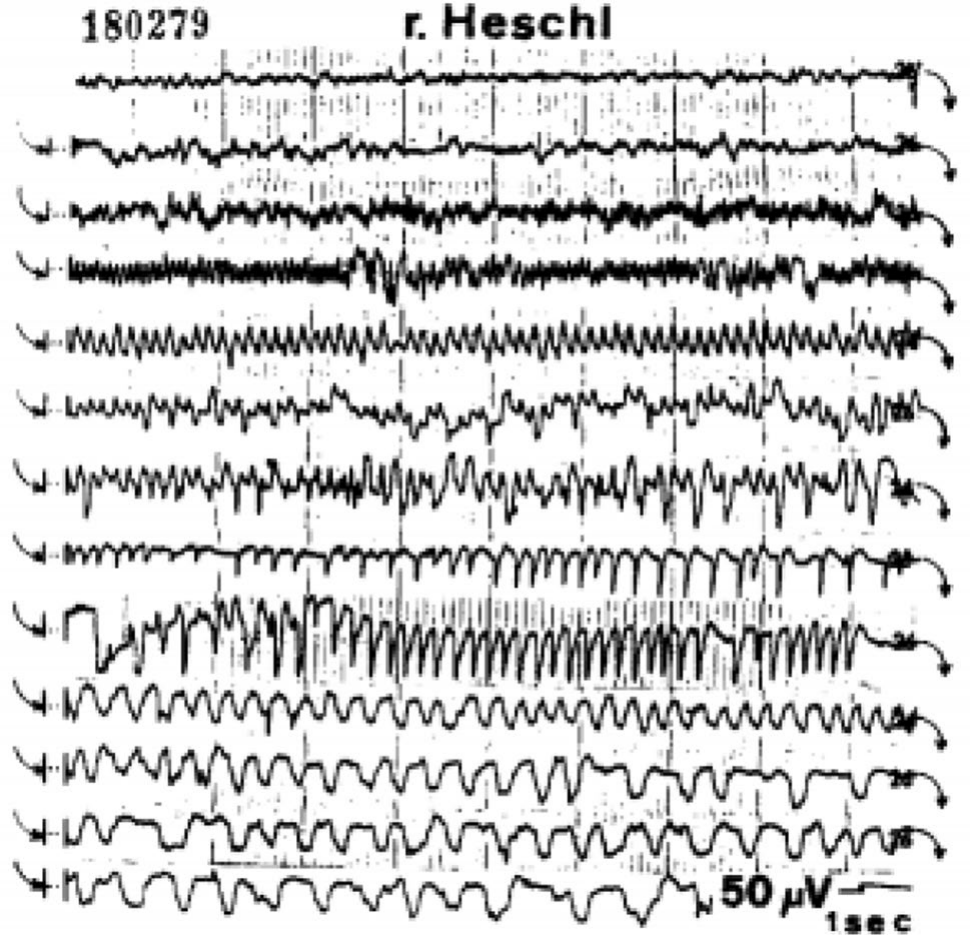
Intracranial EEG showing seizure patterns concomitant with musical hallucinations: single-channel display of a 3-h epileptic discharge from the right gyrus of Heschl, becoming more and more monotonous and showing marked slowing. Typical intervals, spaced approximately 15 min apart. Discharge accompanied by hallucinations of music (Wieser [21])

Wieser described the case of a 22-year-old woman who had undergone continuous surface-EEG monitoring for longstanding epilepsy [21]. Her CT head showed a degree of asymmetric atrophy, particularly around the right Sylvian fissure. She had elaborate psychosensory seizures with dream-state-like recalls, associated with a more pronounced spread of the discharges to lateral temporal cortex, especially to the posterior parts or to the gyrus of Heschl when hallucinations of music became prominent (heard as coming from the right). She initially rated the music (a Portuguese song called Santa Maria) as being of a ‘good quality’, but after 3 h reported that the sound quality became worse. At about the same time the voice of the singer ceased, and the drums became more prominent. After 3 h of hearing ‘the same disc’ in endless repetition, the patient got angry, and told the researchers that she had had ‘enough of our test’. The next day a long-lasting discharge with the same localisation and formal characteristics was recorded, but this time another song (Chico Marvelhoso) was experienced. Auditory signals, and especially music, could influence the spontaneous discharge to some extent (Figs. 2, 3). The patient became seizure-free after epilepsy surgery.

Donnet and Régis described an 81-year-old woman who had sought help for anxious-depressive symptoms, and had experienced musical hallucinations for 2 weeks [29]. These were mostly pieces of opera, which were identical for a period of 2 h at a time (in particular Habanera, the first aria of Bizet’s Carmen) but also military songs (La Madelon and the Marseillaise). Sometimes the music was accompanied by nonverbal auditory hallucinations such as applause or a dog barking. The woman first thought that the neighbours were responsible, but then went on to have multiple episodes of loss of consciousness, followed by confusion, and also complex partial seizures. Although routine blood tests and a first EEG had been normal, another, longer EEG showed sharp delta waves in the left temporal region. During episodes of musical hallucination an intense desynchronisation was seen with a modification of the affective state (crying). An MRI head showed white-matter changes and atrophy. The hallucinations ceased within 48 h. After not tolerating sodium valproate and carbamazepine she was treated with phenobarbital and alprazolam, after which no new episodes were observed for more than a year.

Fénelon et al. [30] reported on a 58-year-old woman with a left parietal metastasis who had started having focal seizures of head-turning, followed by a hallucinated voice, and then familiar songs (by Tino Rossi and Mireille Mathieu). During the weeks after surgery, the hallucinations receded into the background. Although they did not disappear entirely, and the woman had no conscious control over them, phone conversations did diminish their intensity. The EEG was abnormal (some slow activity in the right parietal lobe, without paroxysmal activity).

Sacks described an 88-year-old woman who experienced occasional brief snatches of music a dozen times per day, caused by temporal-lobe seizures due to a small thrombosis or infarction in the right temporal lobe [31]. During the EEG recording, she was able to raise her finger whenever a new episode would begin, coinciding with spikes and sharp waves in the temporal lobes. Eventually the songs disappeared without treatment. Another patient described by Sacks, a woman in her 80s with hearing loss, suddenly heard Easter Parade while in the kitchen, followed, in swift succession, by Glory, Glory, Hallelujah, and Good Night, Sweet Jesus. The songs remained for a year or more, in maddening succession, until one day they gave way to more complex songs, of which there were soon countless ones, sometimes simultaneously, and occasionally accompanied by voices and other sounds. The EEG showed a high voltage and excitability in both temporal lobes, with sharp, spike-like waves, coinciding with the music. On anticonvulsants these musical hallucinations disappeared entirely. A third patient was a male with an astrocytoma and temporal-lobe seizures who heard music during his seizures that he could not identify, even though he found them hauntingly familiar, and he himself was a professional musician. De Maeseneire et al. [32] reported on a 64-year-old female who experienced attacks of bilateral music she had previously heard during yoga sessions. The attacks lasted several minutes, and were accompanied by aphasia and paraesthesia of the right hand as well as the right side of the face. An EEG, recorded during the hallucinatory experiences, showed left frontotemporal periodic lateralised epileptiform discharges, after which a left insular-temporal glioma was found. The music stopped after treatment with carbamazepine 400 mg/day and levatiracetam 1500 mg/day.

We ourselves were consulted by a 34-year-old man who since 18 months had experienced monthly episodes of ‘zoning out’ with a thought in his mind, during which he would hear loud music in his head for a few minutes. At such moments he could not hear anything else. The music was often accompanied by a funny taste or smell, and sometimes by a sad feeling. He had a degree in music, and was surprised that he could not recognise the tunes. Audiology showed no abnormalities, but the MRI head showed a large left temporal glioma.

Mulder and Daly rendered the account of a 40-year-old man with complex episodes during which he leaned to the left, while the right side of his face was pulled up, and his right eyelid twitched [33]. Meanwhile he continually swallowed, licked his lips, and responded briefly in a catchy voice to all spoken questions. His right arm was extended, with the hand half clenched, after which he slouched down in his chair and straightened out his right leg. Episodes like these lasted for 20 to 30 s. About 60 to 90 s later, two fleeting right-sided grimaces were noted. He stated that these were involuntary, and were caused by two sharp, shocking pains. After the episodes, he related that he had heard music ‘like angel voices singing’. A tumour in the middle convolution of the left temporal lobe was found.

Martinez-Perez et al. [34] published the case of a 46-year-old male who had been hearing El Desafio (the theme song of a Colombian TV show) episodically during the last 5 years, mainly in the right ear, lasting for some 10 min, in the absence of any other features. During the months prior to admission, he had complained of bilateral hearing loss, predominantly on the right-hand side, along with personality changes, irritability, and memory deficits. The 72-h video telemetry showed spontaneous left frontotemporal sharp waves and spikes, while the audiogram report showed hearing loss in the right ear. An MRI head (and surgery) showed a left temporoparietal oligodendroglioma. The musical hallucinations disappeared with anticonvulsant treatment before surgery (personal correspondence with the authors).

Schiffter and Straschill described a woman who at age 22 had suffered a left temporo-parieto-occipital bleed that had led to focal seizures with motor, speech, visual, and epigastric manifestations [35]. At age 35 she had had an antipsychotic/fentanyl induction for a cholecystectomy, and on awakening had heard a loud, continuous, and high-pitched sound on the right-hand side. After a phone conversation with her mother she heard Ave Maria (her favourite song) on the same side. Later that night it started alternating with Ave Verum Corpus Natus de Maria Virgine by Mozart, and melodies of the two began to mix. The next morning she heard German folk songs (without music) for hours on end, and Am Brunnen vor dem Tore Da Steht ein Lindenbaum. In the afternoon it was a Viennese waltz, marching music, and old folk songs. The third day she heard classical music from Die Zauberflöte and other opera. When attempting to suppress the music, it became louder and more intensive. She started to have breaks, and began to hear the repeating final part of a Beethoven piece and other symphonies. The sixth day she heard folk songs and motifs of Beethoven’s violin concerto, another favourite of hers. The music stopped when she talked. On day 10, it became slowly intermittent and then disappeared. The last day there were bigger pauses, and simpler sounds, like a monotonous ‘Ha, hi, ha, hi’, and accordion, and a peep. Her valproate and carbamazepine were not changed. An EEG whilst awake and alert and responsive was done, during which the music became softer, going into a high tone, almost an electric sound, before disappearing. The EEG recording showed left anterior and middle temporal rhythmical 8/s slow waves with single or grouped spike/sharp waves that became less pronounced, correlating with the clinical manifestations.

Unfortunately not all case reports of ictal musical hallucinations are so richly detailed. Penfield and Erickson, for example, also described a patient with a meningioma in the lesser wing of the sphenoid that caused musical hallucinations in a man of 59, but without providing much additional information [26]. In some of the other cases it is unclear whether the hallucinations described were aura/ictal or post-ictal phenomena. Thus, Isaacson et al. [36] describe a 60-year-old man who became volatile, and experienced visual and auditory hallucinations as well as paranoid delusions after treatment with oral ciprofloxacin for an ear infection. During an EEG, four clinical seizures were recorded, during which he became unresponsive and stared, with occasional head shaking. There was right temporal 10–11 Hz sharp rhythmic activity for 90 s, followed by focal slowing, and then a return to a normal background rhythm. Despite treatment with phenytoin, the episodes continued for 2 days. He developed palinacusis, as well as episodic auditory hallucinations of voices and music. This gradually disappeared in the following week, and 8 days later the EEG had normalised. Seizures recurred on re-challenge with ciprofloxacin, but not in 2 years of follow-up since.

### Post-ictal musical hallucinations

Like aura phenomena, post-ictal musical hallucinations are reported less frequently than classic ictal ones. One striking example stems from Rennie, about a 63-year-old man with a degree of deafness and likely cysticercosis who ‘felt that a nearby machine sounded like an organ’ (musical pareidolia) [19]. Upon experiencing this, he suddenly thought he was going to die, lost consciousness, and had convulsions lasting 90 min, beginning on the right side of the mouth, and then becoming generalised. The day after admission he had no more seizures, but instead developed auditory hallucinations. At first he heard ‘a deafening singing… a melody without words going up and down the scale like a hymn’. Shaking his head brought some relief. For the next 3 days he experienced almost constant auditory hallucinations, including the Toreador song from Carmen and other French pieces, an English song, nurses talking, a radio programme, and someone playing a piano beside his bed. If a man had been talking to him previously, the singing would be in a male voice, but if previous speech had been in a high-pitched voice, the singing that followed would be in the voice of a woman. The melody was continuous for an hour or more. He then heard a noise like Big Ben and like a mosquito in his right ear, along with rushing in both ears. Neither the seizure, nor the music ever recurred afterwards, but he was left with a continued rushing sound in the right ear. An EEG made afterwards was normal.

We ourselves saw a 51-year-old woman who had had a focal-onset generalised seizure. She remembered waking up in hospital feeling tired. She then intermittently heard fragments of clear music in front of her with both ears, such as songs of her school time, radio, opera, and old Dutch folk songs. She had no control over the music, but did not find it troublesome. When she got home she still heard beat music, and when her husband denied hearing it, she realised it was in her head. The last sounds she heard were those of a clock and a washing machine, and then, after 2 days of hallucinated sounds, it became silent again. She had been started on sodium valproate as well as her usual medication after an ischaemic stroke 6 years previously. She was cognitively well and not depressed (MMSE 29/30, HADS 3). A CT and a previous MRI head showed an old right occipito-temporal bleed and multiple old lacunar infarcts in the pons and basal ganglia. An EEG made after 4 days showed slower activity in the right temporo-occipital areas, with bursts spreading from temporal to frontolateral and parietal areas of isolated or briefer periods of delta activity (0.5–1 s) but no epileptiform activity.

Couper described a 78-year-old woman with severe hearing loss (left more than right) and a previous right occipital stroke, who suffered a generalised seizure, and on recovering from post-ictal aphasia on day three was found singing along with music that she presumed to be coming from a radio in another room [37]. In addition, she heard children’s voices uttering expletives, and also saw children, plus a dog. The music was vaguely familiar, continuous in nature, and mainly orchestral, with interludes of jazz. It was annoying, and interfered with hearing the TV and falling asleep. An EEG on day 17 (whilst hearing music) showed sharp-wave activity with a left posterior temporal focus (whereas a previous EEG had been normal) and, therefore, valproate was added to the phenytoin that she had started on at admission. After increasing the valproate dose on day 32, on day 33 the music became very distant, and on day 34 after a few brief snatches ceased completely.

Hécaen and Ropert described a 48-year-old woman with a previous traumatic brain injury and right-sided hearing loss, who developed acute aphasia that lasted for a day, followed by right-sided musical and verbal auditory hallucinations [38]. The musical hallucinations evolved from bells chiming to a number of different instruments, lasting several weeks. They were suppressed by conversation. The woman had normal arteriography and encephalography (as this was the 1960s), and her EEG showed a slow-wave focus in the left temporal lobe.

We mention these last two case descriptions here, under the heading of ‘Postictal hallucinations’, although they might also have been instances of a focal non-convulsive status epilepticus.

### Interictal musical hallucinations

Interictal phenomena are recognised on the EEG by sharp waves, spikes, spike-wave complexes, and polyspike-wave complexes, which are different from nonspecific, physiologic sharp transients. Roberts et al. [20] reported on a 61-year-old, right-handed woman who had presented with a 2-year history of focal seizures, and a 1-year history of musical hallucinations. Her symptoms had initially begun as episodes of nausea, fatigue, and disorientation that arose without warning, and lasted several minutes. New-onset tinnitus evolved into a persistent, episodic, and bilateral hallucination of music that she recognised, but could not control voluntarily. The music had a seasonal variation, with Amazing Grace and Christmas music in December, for example. A 5-mm aneurysm was found pressing onto the right uncus. Amobarbital injection of the right internal carotid artery stopped the music. During intraoperative corticography, spike discharges were present in this area, which disappeared after clipping the aneurysm—as did the music.

Borelli et al. [39] published the case of a 62-year-old woman with musical hallucinations that were also highly suspect of interictal phenomena. She suffered from right hippocampal-temporal sclerosis, had been seizure-free since 5 years on carbamazepine and clobazam, and had for 3 years heard the background jingle of her favorite television programme, Who Wants to Be a Millionaire. Her perception of the music was so vivid, that more than once she had gone into the living room to see whether the television had been left on. This persisted for at least 6 weeks, during which time the music changed in frequency, intensity, and type (i.e., chansons, Rubinstein, organ music) brought on by perceptions, emotions, and movement. A few months later, the programme on television was replaced by a quiz show, upon which she started to experience the background music of the new programme. Surface EEG, hearing tests, and a psychiatric examination were normal, and she did not experience the music as troublesome.

We ourselves saw a 60-year-old man who had had an ischaemic stroke at age 49, and had later developed focal-onset seizures that were well controlled, initially with phenytoin, and later with levatiracetam 1000 mg/day and valproate 600 mg/day. Our patient had noticed that 3 years after his stroke he had gradually started to hear different types of music, such as the national anthem, Dixieland, and songs by Billy Chant, Take 5, Rubrick, Tunnelbeach, and a country band. The music was not troublesome, and he would often happily hum along. Whenever he switched on the radio, the music would disappear, but otherwise he was unable to influence it. A CT head showed an old right parietal infarction, and an EEG after 6 years showed a right temporal epileptic focus. At follow-up, it showed focal sharp abnormalities with no changes whilst listening to music.

### Musical hallucinations and brain stimulation

Although not strictly part of the group of epilepsy-related phenomena, musical hallucinations induced by invasive and non-invasive brain stimulation are also worthy of our attention in this context. As regards the invasive stimulation techniques, Penfield and Perot did ground-breaking work using unipolar silver electrodes to probe cortical areas directly, while their patients were under local anaesthesia. As the authors described in great detail in their paper, probing the left or right superior temporal lobe from 17 different points produced the hearing of music in 11 patients, who subsequently reported Christmas carols, an orchestra, the theme song of a radio programme, their mother singing, a piano playing, and many other types of music [27].

Among the various types of non-invasive brain-stimulation techniques, ECT (which induces seizures) is also known to be capable of mediating musical hallucinations. Thus, Janakiraman et al. reported on a 93-year-old woman who had had two sessions of ECT, and a few days later complained of hearing musical notes and songs during most of the day, such as Rose in a Garden of Weeds, her favourite childhood song [40]. These non-distressing musical hallucinations persisted up to a few days after the last ECT session. Likewise, Lhermitte and Parcheminey described a 40-year-old female musician with 10 years of progressive deafness, who had received ECT for treatment-resistant obsessions, and after the fifth session immediately heard music that seemed very real [41]. Contrastingly, ECT has also been used as a treatment for musical hallucinations in association with severe depression. In three women, it was administered after the failure of antidepressants. In each case it was highly successful, with one of them leading to remission of the musical hallucinations after as few as two treatments [13].

Cosentino et al. [42] described a 63-year-old male with chronic, moderately severe hearing loss, who suffered a right anterior temporal contusion. He subsequently developed constant musical hallucinations of old Italian songs with a Visual Analogue Scale volume of 5–8/10. An EEG and a neuropsychological assessment yielded no abnormalities. The musical hallucinations did not improve with (subsequently) gabapentin, carbamazepine, risperidone, and paroxetine. He then received daily (5/7) rTMS of 1 Hz (1200 stimuli in 20 min) at an intensity of 90% of the motor threshold. After the first week, the patient reported consistent amelioration and reduction of the musical hallucinations to 1–2 h a day, with an intensity of 2/10. At the end of the 2-week treatment, they had completely disappeared. The same musical hallucinations reappeared after about 4 months, but they no longer troubled him, as they only occurred in environments free of noise, and before falling asleep. A PET scan, 5 months later, showed a normalisation of hypermetabolism on initial PET in right posterior temporal cortex (where TMS was given), but PET hypometabolism at the site of contusion remained.

### Idiopathic musical hallucinations; MEG and EEG

MEG provides a unique capacity to measure as well as localise brain activity. It has been deployed successfully to map activity related to musical hallucinations, albeit with the aid of different protocols. Thus Kasai et al. [43] studied an 88-year-old woman with no obvious cause for her musical hallucinations, who during hallucinatory episodes showed a longer distance of the N100m-response source movement for pure-tone and vowel stimuli than during the absence of musical hallucinations. Vowel sounds without hallucinations were 20 ms (112 versus 92 ms) slower compared to those with hallucinations, but there was no change with pure-tone stimuli. The N100m-response change was located in the right supratemporal gyrus and its vicinity (and none in the left hemisphere). A SPECT scan also showed increased blood flow there, and in the right inferior frontal gyri while she experienced musical hallucinations.

Shinosaki et al. [14] performed MEG in a 78-year-old woman with depression and hearing loss, who had experienced musical hallucinations for 1.5 years. She had an MMSE of 29/30, a normal MRI head, and a normal EEG. What she heard were simple rhythmic sounds, which improved after treatment with donepezil, but returned whenever she was asked to have 20 s of relaxation. When she subsequently listened to a 20-s newscast, they would disappear again. The patient (who was compared to four controls) showed significant desynchronisation—while she experienced the musical hallucinations—in the right transverse gyrus of Heschl and the right planum temporale (in 8–13 Hz), as well as in the right surpramarginal gyrus (in 13–30 Hz). The N100m response also changed with musical hallucinations versus no hallucinations (i.e., in the controls) from the supramarginal to the supratemporal gyrus, bilaterally.

Vanneste et al. [8] studied EEG changes in ten women (with a mean age of 66 years) with continuous musical hallucinations since more than a year, and compared them with ten patients with tinnitus, with similar laterality, loudness, distress level, and hearing loss, and ten healthy control subjects. For the theta- and alpha1-frequency band, a significant increase of power was seen in right auditory cortex in patients with either tinnitus or musical hallucinations. For the beta3 frequency (21.5–30 Hz), increased activity was found in dorsal anterior cingulate cortex, the (para)hippocampal area, and the bilateral insula. In the gamma-frequency band, patients with either tinnitus or musical hallucinations showed increased activity in left and right auditory cortices. Patients with tinnitus (and even more in those with musical hallucinations) had a significant correlation (synchrony) between low and high frequencies in bilateral auditory cortices (vs none in the controls). High frequencies within the beta and gamma range correlated stronger with theta activity in patients with musical hallucinations versus tinnitus. Musical hallucinations versus tinnitus demonstrated increased activity in the left inferior temporal area for alpha1, in dorsal anterior cingulate cortex for beta1/beta2, and in the inferior and medial frontal gyrus for gamma.

Kumar et al. [7] used music as a masker to induce residual inhibition in a 66-year-old woman with a 20-year history of hearing loss, who also suffered from tinnitus, hyperacusis, and (since 15 months) musical hallucinations and palinacusis. The musical hallucinations grew in complexity over time. MEG demonstrated a left-lateralised increase in power, and change in frequencies in four areas, when the musical hallucinations grew in intensity; gamma in the anterior superior temporal gyrus, beta in motor and posteromedial cortex (PMC, an area with a possible role in music perception and memory), and theta/alpha in lateral orbitofrontal cortex (OFC, which was active during unpleasant music). There were no oscillatory power changes between externally presented and hallucinated music.

## Discussion

We reviewed the extant literature on musical hallucinations, and found 983 unique case descriptions, of which 24 had a clear relation to epilepsy. Among them were four detailed descriptions of auras, 14 (classic) ictal, and six post- and interictal cases. Our analysis of these case descriptions allows us to develop hypotheses about the pathophysiology of musical hallucinations attributed to epilepsy, and may perhaps also allow us to extrapolate our findings to musical hallucinations in general, as supported by our review of the EEG and MEG literature in relation with idiopathic musical hallucinations. As we saw, the known etiological groups for musical hallucinations are hypoacusis, brain injuries, epilepsy, psychiatric disorders, and the use of illicit substances or medicines. Epilepsy is held accountable for but a minority of all musical hallucinations, and yet, considering the wide variety of relationships that we encountered, ranging from aura/ictal and post-ictal to interictal phenomena, and even to those that arise post-ECT, we are now in a position to further differentiate the epilepsy group into three, or (if we accept ECT-related musical hallucinations as a separate category) four pathophysiological subgroups. This differentiation invites us to employ the phenomenological characteristics of musical hallucinations, as well as their neurophysiological correlates, to inform an explanatory model of musical hallucinations.

### Top-down and bottom-up prediction errors

An inspiring model for the mediation of musical hallucinations stems from Kumar et al. [7] who take as their point of departure a Bayesian predictive coding hierarchy that allows them to explain how a mismatch between top-down prediction errors (e.g., “what does the brain think it is hearing”) and bottom-up error-prediction errors (“which signal feedback does it get”) can result in spontaneous activity or changes therein. The phenomenology of musical hallucinations in the context of epilepsy does indeed make sense when seen through the lens of such a Bayesian model of top-down and bottom-up (mis-)matches. A case in point is the finding by Wieser, that playing actual music can apparently alter temporal-lobe discharges associated with musical hallucinations [21]. Another relevant instance is the above clinical description of the patient who could not hear any environmental sounds while experiencing musical hallucinations, and had moreover found that changing head position could alter or even stop the music [26]. Two other examples stem from Rennie and from Arieff and Brooks, who described patients who were thus able to influence their musical hallucinations [19, 44]. These findings indicate that musical hallucinations are sometimes amenable to changes in peripheral input, which is in line with the well-documented habit of some patients with idiopathic musical hallucinations to use external music, conversations, and other activities that involve an auditory component to diminish or drown out their musical hallucinations [6].

Another important finding, based on the data we presented above, is that some patients (particularly those with post-ictal phenomena) can move within days from musical hallucinations to other auditory hallucinations, the latter often being less complex in nature, mimicking environmental sounds or taking the shape of tinnitus. This points in the direction of a shared pathophysiological mechanism for musical hallucinations, verbal and nonverbal auditory hallucinations, and tinnitus, thus paving the way for an auditory-network hypothesis that treats these different auditory misperceptions as lying on a continuum while acknowledging their indebtedness to different states of the same network. Incidentally, this approach begs the question of whether the cases categorised above as ‘post-ictal’ might perhaps be forms of post-ictal psychosis (PIP). However, in PIP there is generally a more widespread disturbance of mental functioning than in these cases, and a lucid interval (see criteria as discussed by Trimble et al. [45]). That said, in two of our cases there was indeed a clear transition from aphasia, reminiscent of post-ictal phenomena such as Todd’s paresis, with a gradual transition to simpler auditory hallucinations before petering out.

### Towards a global view of auditory hallucinations

To substantiate a global view of auditory hallucinations, it may be helpful to examine in some more detail the EEG findings in musical hallucinations related to epilepsy. As indicated above, most of the changes reported in the literature were found in (primary and higher) auditory cortex, constituting typical epileptic changes (e.g., spike/wave discharges). However, also reported was rhythmic alpha activity, sometimes followed by slowing or just slowing (often meaning delta/theta activity) [35, 36]. Another finding was desynchronisation, something also found in MEG studies in idiopathic musical hallucinations, in the alpha band in areas of early processing of sound, and in beta in higher areas (i.e., the supramarginal gyrus). Desynchronisation can occur during seizure onset, as can increased power in the beta and gamma ranges (15–40 Hz). Although the meaning and even the measurement of (de-)synchronisation in epilepsy remain controversial issues [46], the findings by Vanneste et al. [8] concerning increased synchronicity between high and low frequencies in auditory cortex in tinnitus and idiopathic musical hallucinations could possibly be seen in this context.

Both Kumar et al. and Vanneste et al. found increased gamma power in the anterior superior temporal plane, i.e., the area that shows increased BOLD activity on fMRI scans during the normal perception of melody, albeit by Kumar et al. [7] in the left, and by Vanneste et al. [8] in the right hemisphere [47]. In addition, the latter group found increased gamma power in primary auditory cortex, bilaterally, while both studies showed increased power in alpha and beta bands in ‘higher’ areas, including strong beta-power increases in premotor cortex. This is consistent with hypotheses suggesting that predictions are transmitted through gamma bands, and prediction errors through beta bands [48].

### Analogies with tinnitus

This model can be expanded by including knowledge of the network pathophysiology of tinnitus, which, as we saw, can be seen as lying on a continuum with that of musical hallucinations. This connection is further supported by the finding that the whitenoise characteristic of tinnitus can sometimes take on a musical quality, thus evolving into musical tinnitus, which is phenomenologically indistinguishable from musical hallucination [49]. In addition, the original noise-type tinnitus can in such cases become less noticeable or appear to be fully absent [50]. One extant case of ECT-induced chronic tinnitus, and one of ictal tinnitus, also underlines commonalities [51, 52].

Mohan et al. [53] studied the EEG of 311 tinnitus patients and 256 control subjects. As they found, the auditory networks in these two groups were distinct in all frequency bands, but substantially overlapped in the gamma-frequency band. In addition, there were differences in network topology in the delta, theta and higher beta bands; in the alpha band by changes in hubs, and in the gamma band by changes in network connectivity. In a different study, Mohan’s group used graph theory, and found a significant increase in connectivity in beta and gamma oscillations, as well as a significant reduction in connectivity in the lower frequencies for the tinnitus group [54]. The parallel processing of long-distance information between delta, theta, alpha1, and gamma frequency bands was significantly stronger in the tinnitus group. While the network reorganised into a more regular topology in the low-frequency carrier oscillations (the top-down prediction), a more random topology was seen in the high-frequency oscillations, which would seem to hint at a deafferentiation-based bottom-up prediction error. In a further study, this group investigated the effects of virtual lesions on the robustness and dynamics of brain networks in patients with and without tinnitus. The tinnitus networks were robust when lesions affected random and rich-club nodes, but they were drastically modified when lesions affected the periphery, especially while targeting feeder hubs [55]. These studies support the model of thalamocortical dysrhythmia (TCD) as an adaptive mechanism to missing auditory input (in case of hearing loss) in tinnitus [56]. In deafferentiation, TCD is characterised by a slowing-down of resting-state alpha to theta activity associated with an increase in surrounding gamma activity, resulting in persisting cross-frequency coupling between theta and gamma activity. In limited deafferentiation, the missing information can be retrieved from the auditory cortical neighbourhood, thus decreasing surround inhibition, which then results in TCD. However, when deafferentiation increases, the TCD might change to persisting parahippocampo-cortical activity, and thus to a pathological theta-gamma rhythm. According to this model, the difference between tinnitus and musical hallucinations relies on differential alpha-band activity in auditory cortex, and on beta activity in dorsal anterior cingulate cortex and the (para)hippocampal area. Abnormal coupling between high (beta/gamma), middle (alpha), and low (delta) frequencies in discrete auditory, parahippocampal, and inferior parietal “hub” regions also correlated with tinnitus in an awake patient undergoing cortical mapping [57]. It is tempting to hypothesise that similar changes (TCD and abnormal coupling) might be at play in the mediation of musical hallucinations, so this is an area worthy of further study.

### Brain lateralisation

Although lateralisation has often been studied in musical hallucinations associated with lesions, we found no obvious pattern in the EEG and MEG studies that might corroborate the dominant involvement of either hemisphere. Perhaps this is due to the fact that the individual variability was high. As indicated by Bernardini et al. [58] functional neuroimaging in 23 patients with musical hallucinations yielded a wide variety of identified areas between and within studies. And yet they also found commonalities, involving the basal ganglia, primary and secondary auditory cortex, the precuneus, and orbitofrontal areas.

The areas identified above play a role in processes that could contribute to musical hallucinations, e.g., increased beta power in dorsal anterior cingulate cortex (an area involved in determining whether an auditory stimulus is perceived consciously or not [59]), filling in missing auditory information [60], and successful auditory memory retrieval [61]. In addition, it is part of the salience network, where auditory attention and comprehension interact [62, 63]. The insula has a function in auditory processing [64]. The changes there might be related to auditory awareness and its attribution to an external sound source, whereas the right inferior frontal area plays a role during musical imagery and retrieval of familiar musical information [65].

### A central role for network dysfunction in musical hallucinations?

A final question to be answered is whether this model of (de-)synchronisation and altered connectivity and coupling in the networks implicated in perceiving music may also be of aid in explaining other types of musical hallucination, i.e., types that are not primarily attributed to epilepsy. If we look at musical hallucinations coinciding with intoxications, this might well be the case. Judging by the literature, the list of implicated pharmacological agents is wide, although especially benzodiazepines, opiates, and dopaminergic and anticholinergic medications appear to play prominent roles, which all, in their own way, alter network frequencies, power and dynamics [6]. Acetylcholinesterase inhibitors, in particular, have been shown capable of drastically diminishing, and sometimes completely alleviating musical hallucinations within days, even in cases where they had been present for 6 years, and in another study for respectively 8 and 20 years [66, 67]. A variety of antiepileptics have successfully been used to treat musical hallucinations in a wide range of etiologies, beyond epilepsy alone [6]. Animal studies show how increasing the dose of the acetylcholinesterase inhibitor, physostigmine, shifts the theta rhythm peak to lower frequencies (3.6–4.9 Hz), and decreases power at 5.7–11.9 Hz. In such cases the power of the beta-1 rhythm (13.8–16.4 Hz) is also strongly suppressed, whereas the power of the beta-2 rhythm (20.3–26.5 Hz) is being increased several-fold [68]. Opiates, on the other hand, slow all frequencies down [69]. Benzodiazepines increase beta and gamma power, while dopamine agonists, as studied in the context of Parkinson’s disease, decrease beta coherence and increase cortical beta power [70, 71]. Sodium valproate and carbamazepine have been successfully used as treatment in idiopathic musical hallucinations; both are known to influence EEG power and synchronisation [72, 73]. We, therefore, hypothesise that pharmacological agents such as these have an effect on musical hallucinations by disrupting or normalising electrophysiological activity in the musical-hallucination network. As a consequence, we consider this a direction worthy of further study, preferably with the aid of individual pre- and post-treatment assessments.

Another possible indicator for the involvement of electrophysiological changes is that many patients with idiopathic musical hallucinations describe how they first arose either on awakening or at bedtime, i.e., during moments of significant frequency and network transitions in the brain. Most patients with idiopathic musical hallucinations describe that their hallucinations are attenuated or even disappear during environmental stimulation (e.g., by the TV, the radio or a conversation; events that are known to alter EEG dynamics in beta and gamma frequencies immediately or with variable latency) [74].

The clinical and neurophysiological phenomenon of residual inhibition (temporary inhibition/suppression after a stimulus) has been studied extensively in tinnitus and experimentally induced in musical hallucinations. Post-ictal clinical and EEG changes can be seen in this light [7, 49]. We have many times observed in idiopathic musical hallucinations that after 40–60 s of quiet in a consulting room the music is perceived. This time course is interesting; not the flick of a switch, but a gradual building up of a new perceptual reality as also seen clinically with neurophysiological correlates in a patient with tinnitus undergoing awake cortical mapping [57].

## Summary

Summing up, musical hallucinations in the context of epilepsy can be divided into three subtypes, comprising aura/ictal phenomena, post-ictal phenomena, and inter-ictal phenomena, and even into four, if we also include post-ECT phenomena. Since our analysis of the phenomenological and neurophysiological data from extant case descriptions indicates that musical hallucinations lie on a continuum with other nonverbal auditory hallucinations, verbal auditory hallucinations, and notably tinnitus, we propose a Bayesian model involving top-down prediction errors and bottom-up error-prediction errors that incorporates findings from EEG and MEG studies to explain the mediation of musical hallucinations from within the auditory network. As we demonstrated, this electrophysiological auditory-network hypothesis also has ramifications for musical hallucinations in their relation to pharmacological substances. We, therefore, suggest that musical hallucinations associated with epilepsy can teach us important lessons on how some other types of musical hallucination are produced, i.e., through alterations in wide-ranging networks, through (functional and structural) disruptions in connections, through abnormal (de-)synchrony between different frequencies, and through changes in their power in a Bayesian predictive coding hierarchy.

## Limitations

The cases of epilepsy in musical hallucinations described above have often no documentation of hearing loss, as well as other incomplete data. The patients have many different causes of their epilepsy, including structural abnormalities. The numbers are small, and there is insufficient data on the influence of pharmacological agents or environmental stimuli on the neurophysiology in patients with musical hallucinations.
